# Premature ovarian insufficiency: A hormonal treatment approach: Number 2 - August 2020

**DOI:** 10.1055/s-0040-1716929

**Published:** 2020-09-08

**Authors:** Cristina Laguna Benetti-Pinto, José Maria Soares Júnior, Gustavo Arantes Maciel, Andrea Prestes Nácul, Daniela Angerame Yela, Ana Carolina Japur Sá Rosa e Silva

**Affiliations:** 1 Departamento de Tocoginecologia, Faculdade de Ciências Médicas, Universidade Estadual de Campinas, Campinas, SP, Brazil; 2 Departamento de Obstetricia e Ginecologia, Hospital das Clinicas, Faculdade de Medicina, Universidade de São Paulo, São Paulo, SP, Brazil; 3 Hospital Fêmina, Grupo Hospitalar Conceição, Porto Alegre, RS, Brazil; 4 Departamento de Ginecologia e Obstetrícia, Faculdade de Medicina de Ribeirão Preto, Universidade de São Paulo, Ribeirão Preto, SP, Brazil

## Key points

- Premature ovarian insufficiency (POI) is characterized by loss of ovarian function before the age of 40 years.
- Diagnosis is based on follicle-stimulating hormone (FSH) levels >25 mIU/mL measured on two occasions (different samples) at least 4 weeks apart.
- POI is suspected in the presence of irregular menstrual cycles or secondary amenorrhea in women before the age of 40 years, or in girls with primary amenorrhea.
- Symptoms of hypoestrogenism are usually present, but this not mandatory.
- Most POI cases are idiopathic, but they may also be due to autoimmune disease, genetic cause, oophorectomy, chemotherapy, radiation therapy (RT), and other less frequent causes.
- POI is of genetic background in about 10% of cases, most commonly in patients with primary amenorrhea.
- Chromosomal analysis (Karyotype) is indicated in non-iatrogenic POI cases. In the presence of Y chromosomal material, patients must be submitted to gonadectomy because of the high risk of gonadal tumours.
- Despite being associated with infertility, spontaneous pregnancy may occur in 5% to 10% of POI cases,
- Good therapeutic practices include providing guidance on a healthy lifestyle and sexual and psychological assessment and follow-up.
- Hormonal treatment is mandatory for all POI-women with no contraindication , and it shall be continued until the usual age of menopause.

## Recommendations

- Upon suspected POI, any hormonal treatment must be suspended at least 60 days before FSH measurement. Diagnosis may only be confirmed after two repeated measurements at least 4 weeks apart.
- Chromosomal analysis (karyotype) must be requested for all non-iatrogenic POIwomen, preferably for those before the age of 30 years.
- As soon as the diagnosis of POI is made, DXA scan is indicated to assess bone mass and aid in establishing therapeutic recommendations.
- Adopting a healthy lifestyle including physical activity and maintenance of proper body weight, as well as having a healthy diet with the an adequate calcium intake and avoiding smoking will aid in the prevention of cardiovascular and osteometabolic diseases.
- Unless contraindicated, the formal recommendation is estrogen replacement therapy with menacme-adjusted doses to improve vasomotor and genitourinary symptoms, maintain bone health, prevent osteoporosis and reduce the risk of fractures, administered until the physiological age of menopause.
- In patients with an intact uterus, progestagen for endometrial protection must be associated with the estrogen therapy, in a cyclic or continuous regimen. There is no evidence thus far to sustain the use of androgens for all POI patients.
- In POI women who may present episodic ovulation and do not wish to risk pregnancy, there is a need for the use of contraception.
- For infertility treatment in POI cases the recommendation is assisted reproductive techniques with donated oocytes.
- Assessments of sexual function, psychological disorder, sleep quality, and quality of life should be incorporated into clinical practice.
- Annual clinical follow-up is recommended to verify patients adaptation and adherence to the proposed hormone replacement therapy. However, screening for cancer (cervix, breast, and colon) and metabolic disease should be conducted under the same indications and periodicity as set for women of the general population.

## Background

POI is a condition caused by loss of ovarian activity before the age of 40 years. 1 It is characterized by irregular menses consisting of prolonged or absent menstrual cycles associated with a reduction in ovarian capacity for producing sex steroids and an increase in gonadotrophins, i.e., a state of hypergonadotrophic hypogonadism. 1

In addition to menstrual disturbance lasting at least four months, POI diagnosis requires elevated FSH levels measured on two occasions at least one month apart. The European Society for Human Reproduction and Embryology (ESHRE) has currently accepted and recommended FSH cutoff value of up to 25 mIU/mL. 1 2

The term “premature ovarian insufficiency” is not universally used. The condition has already been called “early menopause”, “early ovarian failure”, and “premature ovarian failure”. However, its progression is known to be long and variable, with irregular and unpredictable ovulation in 50% of cases and pregnancy in up to 5% to 10%. 3 4 Another variation is “primary ovarian insufficiency”. 1 4 5 Febrasgo suggests the use of the term “premature ovarian insufficiency” and as a preferred terminology.

POI prevalence at 35 years of age is of 0.5% and at 40 years of approximately 1%. 6 Frequency seems to vary according to ethnicity: it is more frequent in women of Hispanic and African-American origin (1,4%) and less frequent in Japanese women (0,5%). 7 POI generally occurs after a normal puberty and regular cycles, but in 10% of cases it manifests as primary amenorrhea. 2

## What are the mechanisms and causes of POI?

Mechanisms involved in POI are either follicular depletion or dysfunction. Follicular depletion is the most common mechanism and may be due to a reduction in the initial number of primordial follicles, an increase in apoptosis (accelerated follicular atresia), or follicle destruction. 8 In follicular dysfunction, the follicle fails to respond to gonadotrophins. This mechanism is often rare and preferably associated with enzyme deficiency (17α-hydroxylase, 17,20-desmolase, aromatase) and receptor mutation (FSH, luteinizing hormone [LH], G protein). 2 Regardless of the quantitative or qualitative nature of the mechanism, clinical manifestations and the risks associated with POI remain the same. Most POI cases are considered idiopathic, i.e., of no determinable cause. 1 2 However, it is important to investigate underlying causes and associated conditions that may impact the patient’s overall health and, in case of pregnancy or family counseling, the possibility of the transmission of inherited conditions. 1 2 The main etiological groups are given below.

## Genetic causes

The most frequent genetic causes are numerical or X chromosome structural abnormalities, such as Turner syndrome and full/partial deletions, translocation, and other abnormalities involving the X chromosome. 2 5 Despite this genetic abnormality profile most frequently presents as primary amenorrhea, it can also manifest as secondary amenorrhea. 9

The fragile X syndrome (the most common cause for inherited mental retardation) is a genetic condition associated with the X chromosome and caused by a mutation of the *FMR1* gene. Women presenting with a premutation of the *FMR1* gene are at an increased risk of developing POI. Despite its accounting for a small percentage of the genetic causes of POI, this abnormality is present in up to 13% of familial cases. It does not cause mental retardation in the patient who carries it (premutation), but it may lead to the gene’s full mutation in the following generation, which will present with full expression of the syndrome. For this reason, the assessment of POI patients, when detected genetic origin, should also include family genetic counseling. 2 5

Autosomal disorders consist of rare syndromes that may be associated with POI; there is no indication for routine investigation. These may include, but are not limited to, galactosemia, mutation in hormone receptors (LH, FSH), blepharophimosis-ptosis-epicanthusinversus syndrome (BPES) and defects in proteins and enzymes involved in steroidogenesis. 2 3 5

## Autoimmune causes - Association with autoimmune disease/autoimmunity

It is estimated that about 20% to 30% of POI cases are associated with autoimmune disease. 2 The most frequently involved organ is the thyroid, with Hashimoto’s thyroiditis affecting 14% to 27% of patients with autoimmune involve-ment. 2 Despite showing fair inferior prevalence, the autoimmune conditions of adrenal insufficiency (Addison’s disease) and type 1 *diabetes mellitus* are also associated with POI; as well assystemic lupus erythematosus, rheumatoid arthritis, pernicious anemia, vitiligo, and Crohn’s disease. 8

## Iatrogenic causes

### Pelvic surgery

Pelvic surgery is the most frequent cause for hormonal deficiency in premenopausal women. 10 POI may be caused by a reduction in ovarian tissue, such as in cystectomy or oophorectomy, or even changes in local blood flow due to surgical procedures such as hysterectomy or tubal ligation (not consensual) and also by local inflammatory processes. 11 )

### Chemotherapy

Many chemotherapeutic drugs are toxic to oocytes and granulosa cells and may cause depletion of the primordial follicles and/or damage to follicle maturation. 12 Regardless of cell cycle stage, cytotoxic alkylating agents are the most frequently associated to gonadal disorders. 13 The mechanism of damage is believed to be a massive destruction of the population of developing follicles. As a consequence, there is a drop in estrogen levels and compensatory elevation in FSH, thereby recruiting a new pool of quiescent follicles that will rapidly be destroyed; this cycle of rapid activation and depletion of the follicular population has been refered to as *burn-out* . 14 This category of drugs includes cyclophosphamide, ifosfamide, dacarbazine, busulfan, melphalan, and chlorambucil. Procarbazine (derived from an alkylating hydrazine) also shows high gonadal toxicity. 13 15 This phenomenon, which may be transitory, is influenced by patient's age, type of chemotherapy drug and administered dose. The older the patient, the higher the probability of developing POI. 8 13

### Radiation therapy

Oocytes are very sensitive to radiation. Ovarian damage depends on the irradiation fieldand total cumulative dose. 13 POI development post-RT depends on the ovarian reserve available pre-irradiation ; thus, ovarian sensitivity to radiation increases with age. 13 16

### Other Causes

Other etiologies of POI—yet of no solid evidence in the literature—are smoking, infections (mumps, rubella, chicken pox, tuberculosis, and malaria), chemicals (such as bromopropane and vinylcyclohexene dioxide), environmental toxins and heavy metals. 11 17

## How to investigate POI?

The diagnosis of POI is based on clinical history and revised measurements of elevated gonadotrophin levels. FSH levels greater than 25 mIU/mL,measured in two different samples with at least four weeks apart in women younger than 40 years old confirms the diagnosis of POI. 1 2 8 Clinically, a menstrual disturbance of oligo/amenorrhea must be present for at least four months, with or without symptoms of hypoestrogenism (for instance, hot flushes). 1 8

## Care of women with POI: What to assess?

The assessment of a POI-women requires a detailed history and physical examination seeking for signs of hypoestrogenism and comorbidities that could point to a possible cause. A detailed personal and family history may raise situations associated with POI, such as autoimmune disease and genetic cause. 2 3

In cases of primary amenorrhea either with or without delayed puberty, signs of genetic abnormality must be investigated (i. e., Turner syndrome stigmata, such as short stature, webbed neck, low hairline, and cubitus valgus). It is also necessary to assess the development of secondary sexual characteristics, especially breasts. 2 8

After excluding the possibility of pregnancy, women presenting with irregular menstrual cycles (oligomenorrhea) or secondary amenorrhea should be assessed for a differential diagnosis considering other menstrual disturbances, such as: polycystic ovary syndrome, hyperprolactinemia, hypothalamic or hypophyseal (hypogonadotrophic) amenorrhea and thyroid disease. 2 8

Women may report symptoms of estrogen deprivation, such as vasomotor (hot flushes) and genitourinary (vaginal dryness, urgency and urinary frequency) symptoms. The more acute the condition, the more significant the vasomotor symptoms will be. Changes in mood, sexuality and sleep pattern may be present and interfere negatively in quality of life. 1 2

## What are the relevant tests in investigating a women with POI?

Some tests are indicated in order of diagnosis confirmation and others may be requested to investigate the cause of POI or to assess the repercussions of hypoestrogenism, as described below.

### Hormonal assessment: 1 3 8

- FSH (mandatory; measurement on two occasions at least four weeks apart. Serum levels >25 mIU/ mL confirms POI diagnosis);

For a differential diagnosis or complementation purposes, the need for the tests bellow should be assessed:

- Prolactin (differential diagnosis);
- Thyroid-stimulating hormone, TSH (differential diagnosis or assessment of an association with an autoimmune thyroid disease);
- Anti-mullerian hormone (AMH): (not-mandatory; serum marker of follicular reserve; highly restricted indication). 5

### Genetic investigation: What to investigate?

- *Karyotype:* Must be requested for all women with non-iatrogenic POI, especially before the age of 30 years. 1 Chromosomal abnormalities are found in approximately 10% of the cases, more frequently in women presenting with primary amenorrhea. 18 19 The presence of a Y chromosome is an indication for gonadectomy due to the risk of tumor, especially gonadoblastoma. 1
- *FMR1* gene premutation workup (fragile X syndrome): Not only is it indicated for an etiological diagnosis, but also especially for family genetic counseling; this workup must be requested after careful counseling to go over the implications of possible results. 1 2 8
- The routine investigation of genetic autosomal abnormalities is not recommended, except upon suspicion of a specific mutation. 1

### Autoimmune disease investigation: What are the necessary tests?

- Thyroid antibodies (TPO antibodies): Indication for POI cases of unknown cause or upon suspicion of autoimmune disease. In case of positive result, annual measurement of TSH is recommended when thyroid disease has still not manifested. In case they are negative, assessment of thyroid function should be conducted under the same indications of women from the general population. 1
- Adrenal antibodies: Some Societies recommend this investigation for POI cases of unknown cause or upon suspicion of autoimmune disease; the authors note these antibodies are infrequently found in women with POI, and this is an investigation of complex interpretation. Despite this being an indirect marker, the presence of adrenal antibodies allows to infer on a possible autoimmunity leading to ovarian damage. If they are identified, the patient must undergo endocrinological assessment (possibility of Addison's disease at a pre-clinical stage). 1 8

The authors point out that the absence of positive antibodies does not exclude an autoimmune disease origin, once this may be due to an untested antibody or a disease remission period. Additionally, the treatments for idiopathic or autoimmune POI are similar and there is no change in therapeutic approach.

### What are the image tests indicated in the POI investigation?

- Bone densitometry: POI is a significant cause of bone loss and osteoporosis. Initial assessment of bone mass by bone densitometry is recommended. Subsequent tests should be carried out depending on these initial results (new evaluations are especially recommended in cases where there is already bone loss and/or osteoporosis), the use or not of hormone replacement therapy (HRT), and to evaluate therapeutic response in cases of osteoporosis. 1 9 19
- Pelvic Ultrasound: Might be indicated for the differential diagnosis of other causes of amenorrhea. 1

## Treatment: What are the goals?

The objectives of POI treatment are symptoms relief and reducing the repercussions of hypoestrogenism. Although vasomotor symptoms (hot flushes) are the main apparent reason for the use of HRT, reasons related to greater morbidity (i.e., bone loss) must be made clear and reinforced to the patient. 19 Psychosocial support should be offered, with special care regarding the reproductive aspects. 1

Estrogen replacement is recommended to maintain bone health, prevent osteoporosis, and reduce the risk of fracture. 1 19 Likewise, the early start of HRT continued until the usual age of menopause has a positive effect on the risk of cardiovascular disease. 1 19 HRT is also beneficial for quality of life and sexual function (Chart 1). 20 18

**Chart 1:** Indication for HRT in women with POI

Indication	Rationale/notes
Vasomotor symptoms	HRT is recommended for treatment of symptoms and improvement of quality of life.
Genitourinary symptoms	Systemic and, if needed, local (vaginal) estrogen for treatment of vaginal dryness, irritation, and atrophy; dyspareunia; and urinary symptoms.
Bone health	Maintenance of bone health, prevention of osteoporosis, and reduction of the risk of fracture.
Cardiovascular health	Reduction of the risk of cardiovascular disease until natural age of menopause.
Sexuality	Systemic and, if needed, vaginal (dyspareunia) use for improvement in sexual function.

Source: Adapted from the European Society for Human Reproduction and Embryology (ESHRE) Guideline Group on POI, Webber L, Davies M, Anderson R, et al. ESHRE Guideline: management of women with premature ovarian insufficiency. Hum Reprod. 2016;31(5):926–37. 1 Webber L, Anderson RA, Davies M, Janse F, Vermeulen N. HRT for women with premature ovarian insufficiency: a comprehensive review. Hum Reprod Open. 2017;2017(2):hox007. 19 Committee on Gynecologic Practice. Committee Opinion No. 698: Hormone Therapy in Primary Ovarian Insufficiency. Obstet Gynecol. 2017;129(5):e134–41. 22

## POI-women care: What general guidance must be provided?

Considering the increased risk for hypoestrogenism-associated diseases, the following general guidance should be provided to women with POI:

- A healthy lifestyle including resisted exercise (weight lifting), no smoking, and maintenance of proper body weight;
- Adequate ingestion of calcium and vitamin D (preferably included in their diet, however, if necessary, patient may use supplements);
- Assessing cardiovascular risks, including blood pressure (at least annually) and lipid panel (every five years);
- HRT regimen may be adjusted based on clinical response and annual reassessment.

## How is estroprogestative HRT prescribed?

Among estrogen options, estradiol is the most physiological option in terms of cardiovascular effect when compared to combined hormonal contraceptives (CHCs). 19 Conjugated equine estrogen choices are not in the ESHRE consensus, however the American College of Obstetrics and Gynecology (ACOG) considers them a valid choice for HRT in women with POI. 19 20 21 22

The most widely used progestagens for HRT in women with POI are medroxyprogesterone acetate (MPA), natural micronized progesterone and norethisterone. The dose will depend on the chosen regimen (continuous or cyclic) and on the dose of estrogen. In cyclic regimens progestagen is administered for 12 to 14 consecutive days/month in higher doses when compared to continuous use. 19 With the exception of the first years of pubertal development, no regimen has been proven more beneficial than the other; the patient must be asked whether they prefer to undergo periodic withdrawal bleeding or not. 19 A levonorgestrel-releasing intrauterine device/system is an alternative to oral progestagen, and given its contraceptive action it may also be attractive to women who do not wish to risk pregnancy, in cases where this might be an eventual risk. 19 20 21 22 Hysterectomized women will not require the association of progestogens duringestrogen replacement, with the exception of cases where there is previous endometriosis. 19

Transdermal estrogen (adhesive patches or gel) avoids hepatic first-pass and is associated, when compared to oral estrogen, to a lower risk of venous thromboembolism (VTE), in addition to showing serum hormonal levels closer to age-related physiological levels. 19 Transdermal delivery is preferred for women showing comorbidities, such as arterial hypertension and obesity, and risk factors for VTE (including, but not limited to, immobilization, surgery and trauma). 19 Women showing genitourinary symptoms during the use of systemic HT may require combined vaginal estrogen to relieve dyspareunia and vaginal dryness. 19 20 21 22

HRT shall be prescribed to endometriosis patients presenting with POI secondary to oophorectomy. Continuous estroprogestative therapy is recommended, even if the patient is hysterectomized. 19

Estrogen dose should be at a physiological level to mimic ovarian hormonal production. In women with an intact uterus, progestagen in continuous or cyclic/sequential regimen must be added for the purpose of endometrial protection. 23 Doses recommended for hormonal replacement in POI are listed in Table 1; for young women, formulations must contain a higher estrogen dose (see adult dose in Table 2), which may be lower in older women with POI. Despite there being a sparseness of evidence in the literature, this Commission suggests the maintenance of HRT containing 2 mg (oral, PO) or 100 mcg/day (transdermal, TD) estradiol combined with progestagen in young women, as considered above; this regimen may be changed according to symptomatology and bone mass response. When symptoms are persistent with these doses, it can be increased until 4mg of oral estradiol.

Generally, HRT has no contraceptive effect. Women who do not wish to get pregnant and are suspicious of no definitive or irreversible loss of gonadal function should use an additional contraceptive method (e.g., barrier methods or intrauterine devices) or insert an intrauterine device associated with estradiol (oral or TD) or replace HRT with CHCs in a continuous use regimen, for which there is recent evidence of a beneficial effect on bone mass. 1 19 26 27 There are reports of women with POI showing better acceptance of contraceptives, which may even improve adherence to hormonal treatment; however, an eligibility criteria assessment is indicated to verify whether there are no contraindications to the use of CHCs (we suggest using the World Health Organization's criteria, for instance).

**Table 1:** Regimens for hormone replacement therapy in POI

Drug	Dosage	
Estrogen	Continuous use	
Estradiol (17β-estradiol)	100–200 mcg/day TD (adhesive patch, percutaneous gel)	
Micronized estradiol	1–4 mg/day, oral	
Estradiol valerate*		
Conjugated equine estrogen*	0.625–1.25 mg/day oral	
Progestagen	Continuous use	Cyclic/sequential use
Medroxyprogesterone acetate	2.5–5.0 mg/day, oral	10 mg/day
Norethisterone	0.5–1 mg/day, oral	–
Micronized progesterone	100 mg/day	200 mg/day
Levonorgestrel-releasing intrauterine system (LNG-IUS)	LNG-dosedependent duration	–

Source: Adapted from the Committee on Gynecologic Practice. Committee Opinion No. 698: Hormone Therapy in Primary Ovarian Insufficiency. Obstet Gynecol. 2017;129(5):e134–41. 22 Steingold KA, Matt DW, DeZiegler D, Sealey JE, Fratkin M, Reznikov S. Comparison of transdermal to oral estradiol administration on hormonal and hepatic parameters in women with premature ovarian failure. J Clin Endocrinol Metab. 1991;73(2):275–80. 24 Popat VB, Vanderhoof VH, Calis KA, Troendle JF, Nelson LM. Normalization of serum luteinizing hormone levels in women with 46,XX spontaneous primary ovarian insufficiency. Fertil Steril. 2008;89(2):429–33. 25

TD: Transdermal; PO: Oral. *Not among ESRHE's recommendations.

### How to monitor and follow up women with POI?

HRT patients must undergo periodic follow-up, with special care towards treatment adherence. 19 There is no need for periodic hormone measurements for the purposes of monitoring treatment. 1 22

Tests for cancer screening (cervix, breast, and colon) must be conducted under the same indications and periodicity of women from the same age but without POI. 19 Despite controversial results about the increased risk of breast cancer among women using CHCs, this association has not been seen in women with POI undergoing HRT until the natural age of menopause. 19 22

### Up to what age should HRT be continued?

HRT is recommended to be continued until the usual age of menopause, i.e., around 50 years of age. Continuation should be discussed with the patient considering their clinical condition, the presence or absence of symptoms, and risk-benefit relationship. 19 22

## What are the evidence for androgenic replacement in POI?

Despite controversial results in the literature, many women with POI may present low serum testosterone levels when compared to non-POI women of the same age. 24 25 27 28 29 30 31 Although this testosterone deficiency seems to contribute to the manifestation of POI, there is not enough evidence to recommend routine testosterone administration or replacement therapy in these patients. 22 32

Testosterone can be currently prescribed for women with POI diagnosed with hypoactive sexual desire disorder with no contraindication. 33 34 According to the literature, transdermal testosterone adhesive patches delivering 300 mcg/day are associated with an improvement in sexual function, but these are not commercially available in Brazil, being obtained through handling pharmacy. 35 36 37 Evidence suggest that testosterone levels should be used for three to six months with clinical and laboratory reassessment of serum testosterone levels (to verify whether the dose is not supraphysiological). 33 In case there is no clinical response after six months, therapy should be suspended. 33 In case treatment is continued, a clinical (hyperandrogenism signs) and laboratorial (measurement of testosterone levels) reassessment should be conducted every six months. 33 The patient must be informed that there are no safety data on the use of testosterone for over 24 months, as well as there are no approved formulations commercially available in Brazil. 1 33

## How to induce sexual secondary characteristics in girls with hypergonadotrophic hypogonadism during puberty? 24

Induction of puberty in POI patients is conducted with low doses of isolated estrogen gradually increased over the course of two to three years. Progestagen should be combined in a cyclic regimen upon the first menstrual period or two years after the beginning of estrogen therapy.

The initial 17β-estradiol dose is 12,5 mcg/day (TD adhesive patch) or 0,5 mg/day (PO). Alternatively, conjugated equine estrogen at a dose of 0.3 mg/day may be administered. Estrogen must be increased every 6 to 12 months until the adult dose is reached, after two to three years.ee (Table 2). 38

**Table 2:** Hormone replacement therapy for induction of puberty and its maintenance in adult life

**Estrogen**			
**Drug**	**Estradiol TD (mcg/day)**	**Estradiol PO (mg/day)**	**Conjugated estrogen (mg/day)**
Initial dose	12.5	0.5	0.3
Dose gradual increase (6–12 month interval)
25 50 100	0.5 1.0 1.5	0.625
Adult dose	100–200	2.0–4.0	1.25
**Progestagen**			
**Drug**	**Micronized progesterone PO (mg/d)**	**Dydrogesterone (mg/d)**	**Medroxyprogesterone acetate (mg/d)**
Dose (cyclic use—12–14 days/month)	100–200	5–10	5–10

Source: Adapted from Sá MF, Benetti-Pinto CL. Insuficiência ovariana prematura. Federação Brasileira das Associações de Ginecologia e Obstetrícia (FEBRASGO), São Paulo, 2018. (Protocolo FEBRASGO - Ginecologia, no. 43/Comissão Nacional Especializada em Ginecologia Endócrina). 38

## How to manage the reproductive issues?

In vitro fertilization with donor oocytes is the treatment of choice for women with confirmed POI who wish to get pregnant. 8 Patients with Turner syndrome should undergo previous cardiovascular assessment, once pregnancy is relatively contraindicated for these patients (risk of aortic rupture). 8 39 40

## Final considerations

Once POI diagnosis is established, the patient should receive clear guidance on all repercussions caused by early hypoestrogenism, including compromised fertility. Prolonged estroprogestative HRT is the treatment of choice for POI. It must be individualized according to age, clinical manifestation, and metabolic changes and the patient's preferences must be considered. HRT should be continued at least until 50 years of age, and there is no indication for follow-up with measurement of FSH or estradiol levels. Evidence relative to HRT in women with usual menopause cannot be directly transposed to women with POI. The following general guidance should be provided: adopting a healthy life style in terms of diet and physical exercise and avoiding smoking. Mammograms and oncotic colpocytologies should be conducted under the same indications for women of the general population, while bone densitometry should be considered in the diagnosis of POI with individualized repetition. The aim of POI treatment is to offer an overall improvement in physical, mental and sexual health while simultaneously promoting quality of life.

National Specialized Commission on Gynecological Endocrinology of the Brazilian Federation of Gynecology and Obstetrics Associations (FEBRASGO)

President:

Cristina Laguna Benetti Pinto

Vice-President:

Ana Carolina Japur de Sá Rosa e Silva

Secretary:

José Maria Soares Júnior

Members:

Andrea Prestes Nácul

Daniela Angerame Yela Gomes

Fernando Marcos dos Reis

Gabriela Pravatta Rezende

Gustavo Arantes Rosa Maciel

Gustavo Mafaldo Soares

Laura Olinda Rezende Bregieiro Costa

Lia Cruz Vaz da Costa Damásio

Maria Candida Pinheiro Baracat Rezende

Sebastião Freitas de Medeiros

Tecia Maria de Oliveira Maranhão

Vinicius Medina Lopes
